# Predicting Ischemic Stroke Outcome Using Deep Learning Approaches

**DOI:** 10.3389/fgene.2021.827522

**Published:** 2022-01-24

**Authors:** Gang Fang, Zhennan Huang, Zhongrui Wang

**Affiliations:** Institute of Computing Science and Technology, Guangzhou University, Guangzhou, China

**Keywords:** machine learning, ischemic stroke, deep learning, IST, IS outcome

## Abstract

Predicting functional outcomes after an Ischemic Stroke (IS) is highly valuable for patients and desirable for physicians. This facilitates physicians to set reasonable goals for patients and cooperate with patients and relatives effectively, and furthermore to reach common after-stroke care decisions for recovery and make exercise plans to facilitate rehabilitation. The objective of this research is to apply three current Deep Learning (DL) approaches for 6-month IS outcome predictions, using the openly accessible International Stroke Trial (IST) dataset. Furthermore, another objective of this research is to compare these DL approaches with machine learning (ML) for performing in clinical prediction. After comparing various ML methods (Deep Forest, Random Forest, Support Vector Machine, etc.) with current DL frameworks (CNN, LSTM, Resnet), the results show that DL doesn’t outperform ML significantly. DL methods and reporting used for analyzing structured medical data should be developed and improved.

## Introduction

Stroke is one of the leading causes of death and permanent disability in the last 20 years globally (Global Burden of Disease Collaborative Network, 2018; World Health Organization, 2018). In China, the number of patients diagnosed with stroke each year is approximately 2 million, and the mortality rate is 11.48% (Chen et al., 2017). Stroke is mainly subtyped into ischemic (85%) and hemorrhagic types (15%) (Caplan, 2016). IS occurs when a cerebral artery is blocked (Park, 2017). Long-term physical disabilities after IS can create enormous mental and financial burdens for families and society. Proper exercise and early rehabilitation definitely improve recovery of patients and reduce disabilities (Veerbeek et al., 2011). Predicting a patient’s functional outcomes precisely after a stroke will help physicians in managing an appropriate long-term plan for early rehabilitation. In addition, it guides clinicians in setting realistic goals, provides accurate information to patients and their caregivers, and facilitates the creation of an early discharge plan (Veerbeek et al., 2011). Now, endovascular treatment (EVT) is widely used for IS. Accurate prediction of functional outcomes and reperfusion may potentially improve stroke care, as it can guide selecting the most beneficial treatment option for the individual patient: to perform or to refuse EVT. Recently, clinical variables and radiological image biomarkers are utilized in studies on outcome prediction strategies in ischemic stroke patients after EVT (Venema et al., 2017; Van Os, 2018). More works have been devoted to predicting functional outcomes after stroke (Stinear, 2010; Meyer et al., 2015; Lin et al., 2020). Several medical communities have created and developed scores and methods that can predict the patient’s functional outcomes after a stroke effectively by only using data readily collected at admission (Ntaios et al., 2012; Hilbert et al., 2019). The score statistically analyzes the data and identifies the most relevant predictors from a set of covariates selected by domain experts. The method uses deep learning to predict the functional outcome of patients with acute IS after EVT. Recently, machine learning methods have been ubiquitously used to solve complex problems in many scientific fields, especially in medical science. Medical diagnosis and prognosis prediction are fulfilled in this way (Lin et al., 2018; Van Os, 2018; Debs et al., 2020; Fang et al., 2020).

Recently, DL frameworks have attained great success in various applications, particularly in image processing and natural language processing (NLP) (Hinton et al., 2012; Krizhenvsky et al., 2012), leading to the hot wave of DL (Goodfellow et al., 2016). Though DL frameworks are powerful, they have apparent deficiencies. For example, large scale training data is always required for training, restricting the direct application of DL to tasks with smaller scale data. It is well known that DL is a supervised learning. But nowadays the data of many real tasks are still not sufficiently and correctly labeled due to the high cost of labeling. Because of this, DL frameworks always perform inferiorly in tasks with poor quality data. DL frameworks, especially modern deep neural networks, always possess too many hyper-parameters, and careful tuning of them directly can mainly influence the learning performance of DL. Recently, it is used to diagnose and predict prognosis in the clinical medical field (Ge et al., 2019; Hilbert et al., 2019; Debs et al., 2020). But it is seldomly used to analyze structured clinical medical data. In this paper, currently used DL frameworks are tested to predict stroke outcomes. Furthermore, several ML methods, especially Deep Forest (DF) (Zhou and Feng, 2019), are used to analyze IST dataset and are compared with several DL frameworks. The DF is proposed based on gcForest (multi-Grained Cascade Forest), which is a novel ensemble method of decision tree. This method generates a deep forest ensemble, with a cascade structure which enables gcForest to do representation learning (Zhou and Feng, 2019). Its representational learning ability can be further enhanced by multi-grained scanning when the inputs are with high dimensionality, potentially enabling gcForest to be contextual or structural aware (Zhou and Feng, 2019). In their experiments, the training time cost of DF is smaller than that of DL; even so, DF attains highly superior performance to DL. Herein, the DF and other ML methods are compared with DL to analyze structured clinical medical data. The results show that there are no evidences of superior performance of DL over ML.

## Materials and Methods

### Data

The data used in this paper is The International Stroke Trial (IST) dataset. The IST, including the pilot phase between 1991 and 1993, was conducted between 1991 and 1996 and is a large, prospective, randomized controlled trial, with 100% complete baseline data and over 99% complete follow-up data. The objective of the trial is to know whether early administration of aspirin, heparin, both, or neither influenced the clinical course and outcome of acute IS (Sandercock et al., 2011). The dataset analyzed in this study is downloaded from the IST website. Patients in this trial are identified only by an anonymous code. They were treated more than 20 years ago, and many have died. Hospitals are also identified by an anonymous code. There are no identifying data such as name, address, or social security numbers appearing. Patient age has been rounded to the nearest whole number. Thus, usage of the dataset definitely can’t present material risk to confidentiality of patients.

The following baseline data: time from onset to randomization, gender, age, aspirin administration within 3 days prior to randomization, systolic blood pressure at randomization, presence or absence of atrial fibrillation (AF), level of consciousness, and neurological deficit, are all included in the dataset. Neurological deficits are classified as one of the Oxfordshire Community Stroke Project (OCSP) categories: posterior circulation syndrome (POCS), partial anterior circulation syndrome (PACS), total anterior circulation syndrome (TACS), and lacunar syndrome (LACS). A total of 19,435 patients from 467 hospitals in 36 countries are randomized within 48 h of symptoms onset, of whom 13,020 take a CT scan before randomization, 5,569 are first scanned after randomization, and 846 were not scanned at all. Entries with missing data are deleted, with 18,128 entries left. We exclude patients who are not finally diagnosed as IS. The variable of 6-month outcome is taken as a target. It is represented as 1-dead, 2-dependent, 3-not recovered, 4-recovered, and 8 or 9-missing status. The entries of 6-month outcome with missing status are also deleted. Six-month outcome of 2-dependent and 3-not recovered are merged as one category (not recovered) due to their similarity, and then the target includes three categories (0-*dead*, 1-*not recovered*, 2-*recovered*). At last, 16,403 patients are left. The data of these 16,403 patients finally diagnosed as IS are used to predict the outcome of IS using ML and DL.

## Methods

This paper investigates the ability of some supervised ML methods to predict IS outcomes. Classic ML methods such as support vector machine (SVM) (Cristianini and Shawe-Taylor, 2000), random forest (RF) (Liaw and Wiener, 2002), and deep forest (DF) (Zhou and Feng, 2019) are explored for comparison to DL frameworks such as convolutional neural network (CNN) (LeCun et al., 1998), long- and short-term memory network (LSTM) (Hochreiter and Schmidhuber, 1997), and residual neural network (Resnet) (He et al., 2016). Developing logistic regression models is the usual approach to analyze the stroke outcomes; however, an alternative of ML methods has been proposed, particularly for large-scale and multi-institutional data. The prominent advantage of ML is that it can easily incorporate newly available data and improve prediction performance (Hamed et al., 2014). Nowadays, DL frameworks are prevalent and succeed in the field of image processing and natural language processing (NLP). In this paper, classical ML methods are compared to popular DL frameworks to exhibit their respective performances.

The workflow of the study consists of three sections. Firstly, features collected at the beginning of and on 14 days of randomization in the refined IST dataset (including 16,403 patients) are used. Features, such as date and comments, are removed manually (features are definitely not related to IS outcome). Six-month outcome is kept as the target feature in the dataset. Features that overlap with 6-month outcome are deleted manually. Then, 50 features are kept and used. These features are utilized to predict long-term prognosis (6-month outcome) of acute IS. Based on previous research (Fang et al., 2020), feature selection carried out using recursive feature elimination with cross-validation (RFECV) don’t eliminate explicitly less important features in the whole IST dataset. Thus, all initially chosen features are used to predict. Secondly, a simple CNN framework which consists of three convolutional layers and two fully connected layers is built, and the first convolutional layer is one dimensional convolution. The used LSTM framework is a two-layer LSTM with just one direction and added into a one-dimensional convolutional layer before it. The last layer of the LSTM framework is a fully connected layer. The Resnet lacking bottleneck blocks which consists of eight residual blocks is also added into a one-dimensional convolutional layer as the first layer. This manipulation allows these DL frameworks to accept and process structured clinical medical data, such as IST. ML methods (SVM, RF, Multinomial-Naïve-Bayes, AdaBoost, and DF) are carried out immediately to compare with these DL frameworks. The SVM classifier use linear kernel (with the parameter max_iter = 10,000), and the other ML methods are carried out with default parameters. To implement these methods for this study, we use the libraries of scikit-learn 1.0.1 (Pedregosa et al., 2011) and PyTorch neural networks API (PyTorch., 2021). Thirdly, all these methods are implemented for comparison in predicting accuracy and other metrics.

## Results and Analysis

**FIGURE 1:**
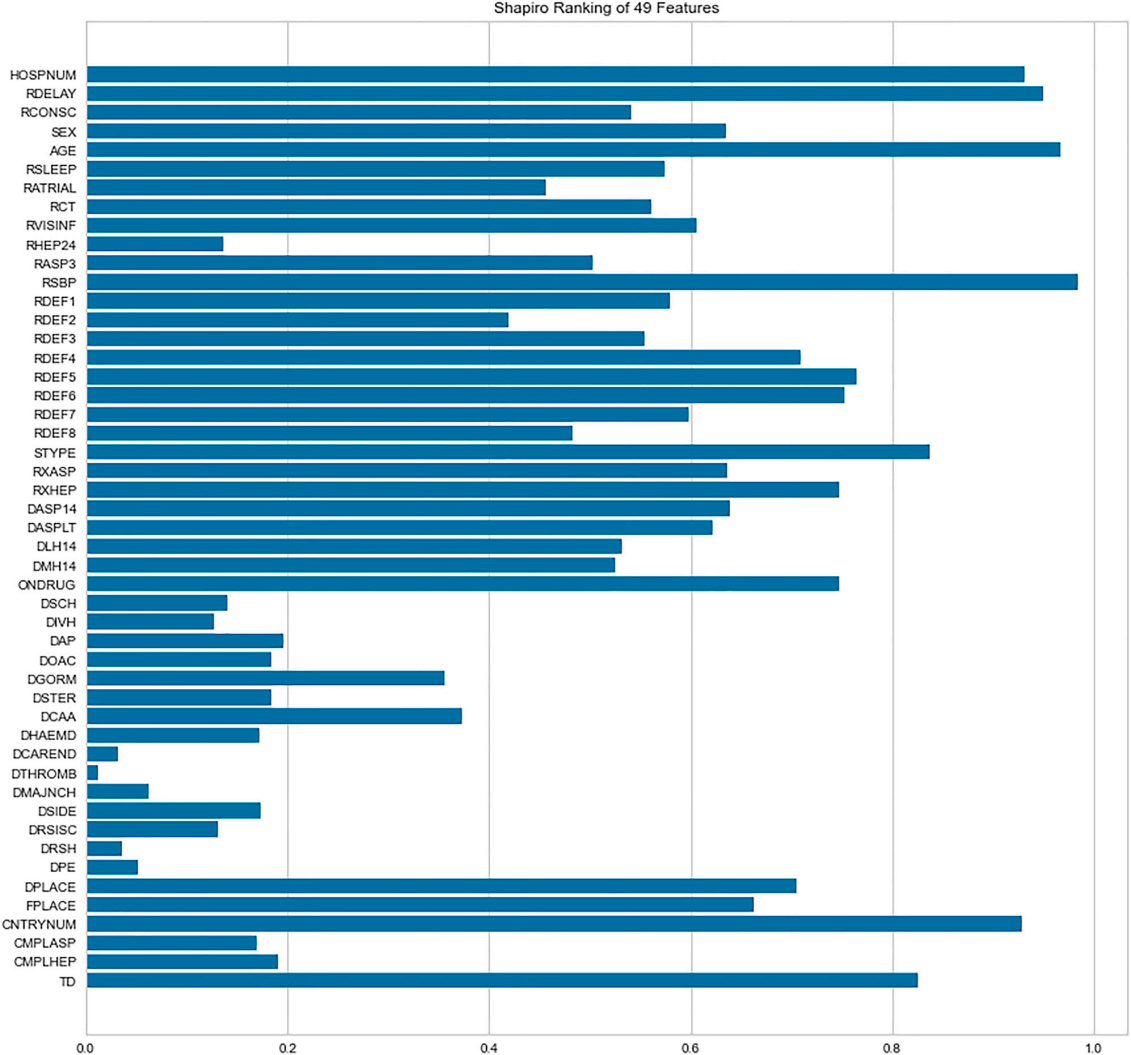
Importance of features ranked by Shapiro-Wilk algorithm.

**FIGURE 2:**
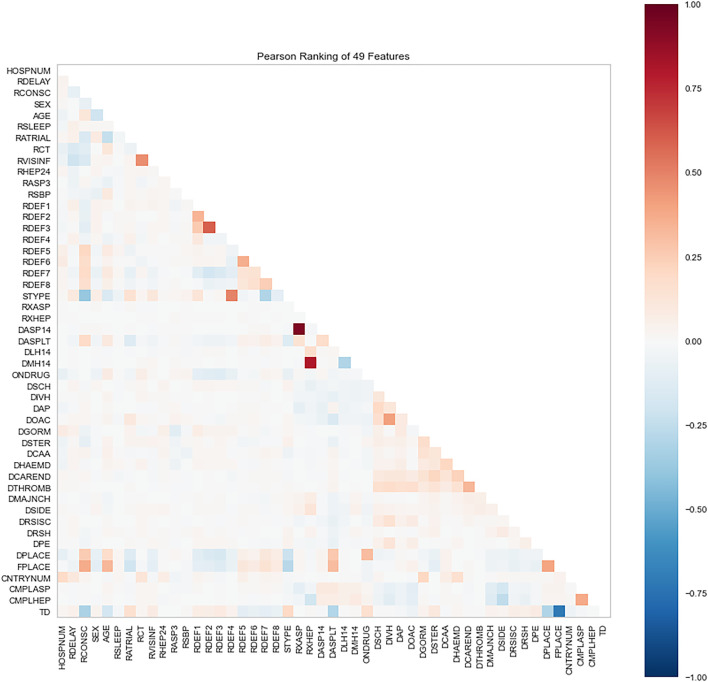
Pearson correlations between features in the dataset.

Because we only consider IS, 50 features are initially selected in the data of all kept 16,403 patients. The feature of 6-month outcome (OCCODE) is kept as target (including 3 categories: 0-*dead*, 1-*not recovered*, 2-*recovered*). The other 49 features include CNTRYNUM, HOSPNUM, SEX, AGE, DPLACE, FPLACE, RDELAY, RCONSC, RATRIAL, RSLEEP, RASP3, RSBP, RXASP, RXHEP, DASP14, DASPLT, RCT, RVISINF, DLH14, DMH14, neurological deficit symptom (RDEF1, RDEF2, RDEF3 … … , etc.), STYPE, ONDRUG, DCAA, DOAC, TD, etc. Readers can be referred to Supplementary Materials for the detailed explanation of these features. Shapiro-Wilk algorithm is used to rank the importance of these features, and Pearson correlations between features are analyzed too. Shapiro-Wilk algorithm is a normal distribution assessing algorithm that regard the instances with respect to the feature, which is improved by Royston to process large data (Shapiro and Wilk, 1965; Royston, 1982). Except OCCODE, the other 49 features are ranked by the algorithm (Figures 1, 2).

The Shapiro-Wilk results show that DTHROMB (Thrombolysis) and DCAREND (Carotid surgery) are the two least important features. The reason for this is that these therapies were seldom carried out in the 1990s. The Pearson analysis shows that high correlations between features are not common in the dataset. The highest correlated features are DASP14 (Aspirin given for 14 days or till death or discharge) and RXASP (Trial aspirin allocated) which are related to aspirin usage. The second highest correlated features are RXHEP (Trial heparin allocated) and DMH14 (Medium dose heparin given for 14 days or till death/discharge) which are related to heparin usage. After this all these 49 features (except OCCODE) are adopted to predict the outcome of IS using ML and DL.

**FIGURE 3:**
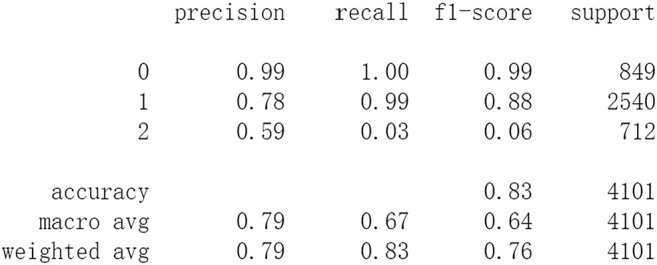
Performance of CNN after 5 epochs of training.

**FIGURE 4:**
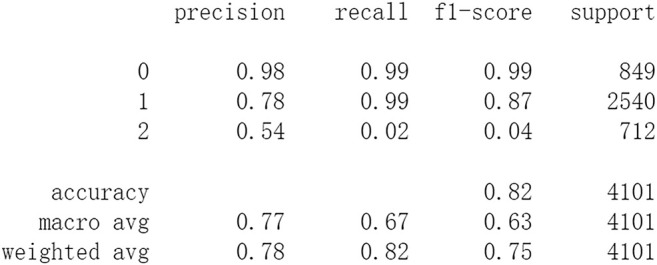
Performance of LSTM after 5 epochs of training.

**FIGURE 5:**
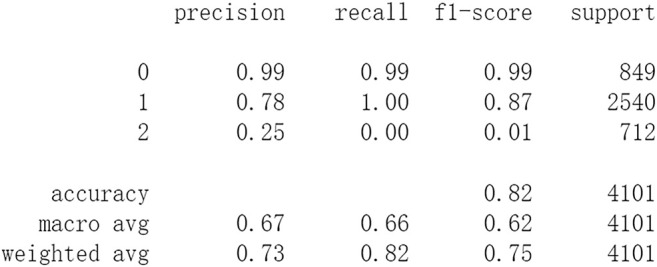
Performance of Resnet after 5 epochs of training.

Firstly, all selected 49 features of the IST dataset which consists of 16,403 patients are processed by DL frameworks. The dataset is divided into training set including 12,302 patients and test set including 4,101 patients randomly. When processed by CNN, 5 epochs of training are carried out and attain an accuracy of 0.826 in test set. Other metrics including precision, recall, and f1-score are also considered (Figure 3). When processed by LSTM, 5 epochs of training are also carried out and attain an accuracy of 0.821 in test set. Other metrics are shown in Figure 4. When processed by Resnet, 5 epochs of training are carried out and attain an accuracy of 0.821 in test set. Other metrics are shown in Figure 5. In this study, all DL frameworks are trained with fewer epochs because more epochs of training lead to overfitting.

**FIGURE 6:**
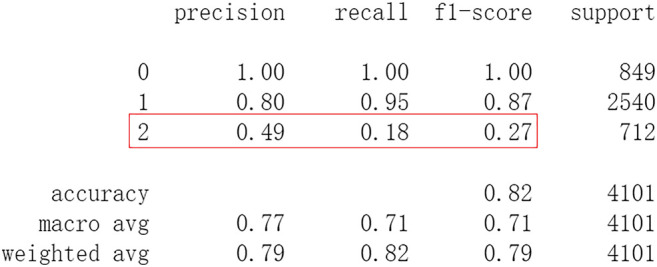
Performance of DF.

**FIGURE 7:**
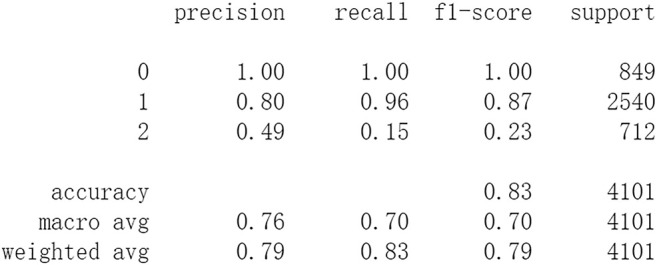
Performance of RF.

**FIGURE 8:**
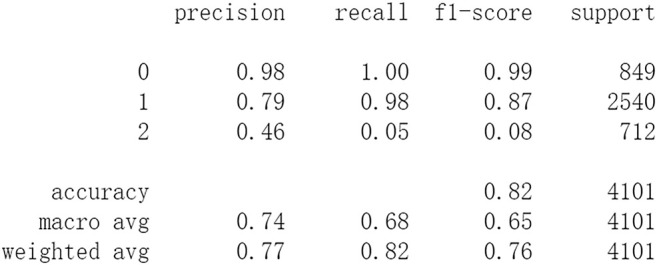
Performance of SVM.

After this, test sets including all 4,101 patients are processed by ML approaches. First by DF, it attains an accuracy of 0.824 in test set. Other metrics including precision, recall, and f1-score are also considered (Figure 6). The performances of SVM and RF are showed in Figures 7, 8. For performances of other ML methods, readers can be referred to Supplementary Materials for more details.

The results show that DL frameworks don’t outperform ML methods in any aspects when predicting IS outcomes in IST dataset. On the contrary, ML methods, especially DF, outperform DL in predicting IS outcomes of *recovered*. It attains a higher precision, recall, and f1-score in predicting the outcomes of *recovered* (represented as 2, Figure 6). All methods, especially DL, don’t work well in predicting the outcomes of *recovered*. The reason of this lies in the heterogeneity of data in this category. In other words, there are more variables that can exert influence on the recovery of IS patients.

**FIGURE 9:**
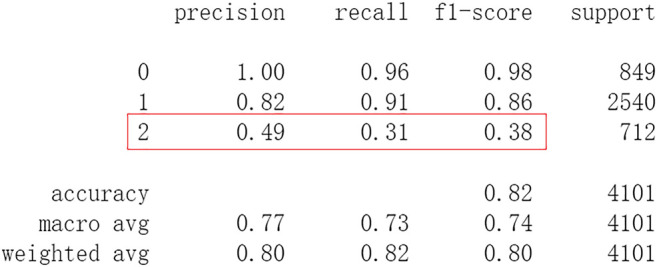
Performance of CNN with 44 features after 20 epochs of training.

**FIGURE 10:**
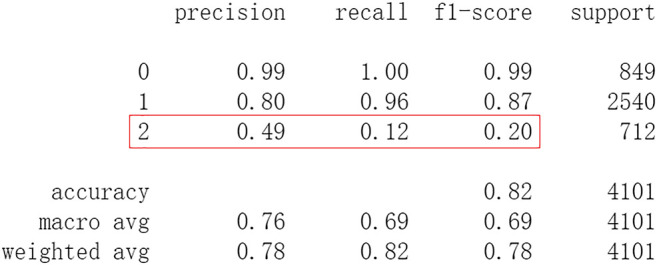
Performance of LSTM with 44 features after 20 epochs of training.

**FIGURE 11:**
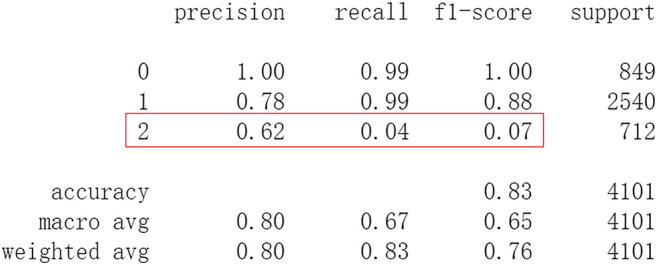
Performance of Resnet with 44 features after 20 epochs of training.

Based on previous Shapiro-Wilk analysis, the less important features whose Shapiro-Wilk ranking value is less than 0.1 are eliminated. These features include DTHROMB (Thrombolysis), DCAREND (Carotid surgery), DRSH (Recurrent stroke within 14 days, Haemorrhagic stroke), DPE (Other events within 14 days, Pulmonary embolism), and DMAJNCH (Major non-cerebral haemorrhage). Then 44 features are left for predicting the outcomes of IS. The predicting performances of DL frameworks are compared to ML methods with these features. When processed by CNN, after 20 epochs of training it attains an accuracy of 0.817 in test set. Other metrics including precision, recall, and f1-score are also considered (Figure 9). After 20 epochs of training LSTM attains an accuracy of 0.823 in test set. Other metrics are shown in Figure 10. After 20 epochs of training Resnet attains an accuracy of 0.827 in test set. The accuracy doesn’t decrease because the eliminated 5 features are less important and not related to the 6th outcome. Other metrics are shown in Figure 11.

**FIGURE 12:**
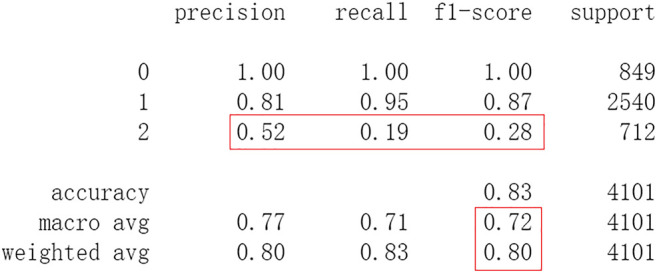
Performance of DF with 44 features.

**FIGURE 13:**
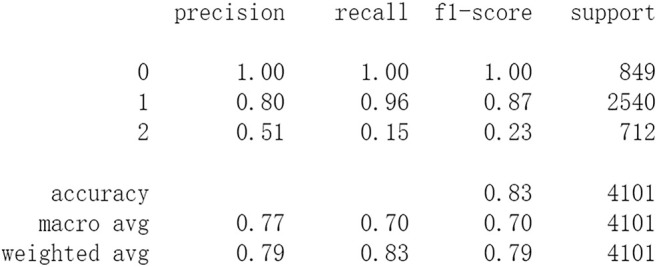
Performance of RF with 44 features.

**FIGURE 14:**
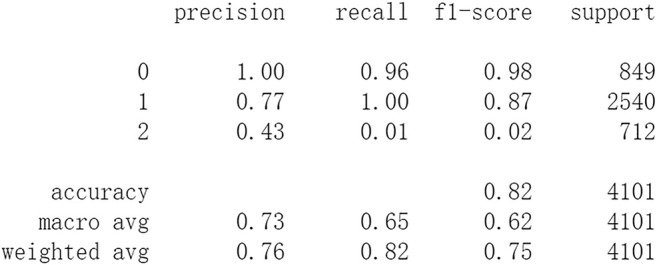
Performance of SVM with 44 features.

Subsequently, test sets including all 4,101 patients with 44 features are processed by ML approaches. DF attains an accuracy of 0.828 in the test set. Other metrics including precision, recall, and f1-score are considered (Figure 12). The performances of SVM and RF are shown in Figures 13, 14. For performances of other ML methods, readers can be referred to Supplementary Materials for more.

**FIGURE 15:**
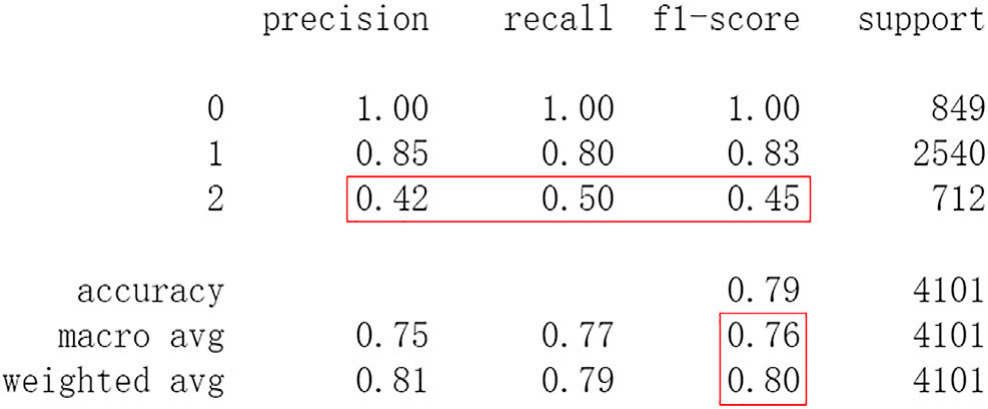
Performance of Resnet with 44 features after 100 epochs of training.

The results show that there is no decrease in predicting performance using both ML and DL after eliminating the five least important features. But compared to previous results, after 20 epochs of training Resnet attains a higher precision than before in predicting the outcomes of *recovered* with 44 features (represented as 2, Figure 11). Considering this observation, more epochs of training are carried out to attempt to explain this. After 100 epochs of training, the predicting accuracy of Resnet is 0.791 in test set. But it gets higher recall and f1-score than before in predicting the outcomes of *recovered* (Figure 15). After 500 epochs of training, the predicting accuracy of Resnet is 0.794 in test set and the other two DL frameworks overfit (accuracy of CNN and LSTM is 0.740 and 0.769 respectively). But there are no increases in recall and f1-score when predicting the outcomes of *recovered* (represented as 2, Supplementary Figure S1). After 100 epochs training of Resnet, the overall predicting accuracy decreased. But macro and weighted average f1-score increased (Figure 15) and are better than before (Figures 3–5, Figures 9–11). Macro and weighted average f1-score are an important index for performance of multi-classification tasks. It is suggested that Resnet will work better when trained appropriately, but it doesn’t outperform ML methods especially DF significantly in this case (Figures 12, 15). When trained 500 epochs, it starts overfitting (Supplementary Figure S1). For more information readers can be referred to Supplementary Materials.

## Discussion

In this study, classic ML algorithms and current DL frameworks are adopted to predict the outcomes of IS in IST dataset. Both methods attain considerable accuracy. The performances of ML and DL are also compared. The results show that adapted DL frameworks don’t outperform ML in predicting capability, although Resnet raised the weighted average f1-score after trained by 100 epochs (Figure 15). The main reason of this lies in that the used DL frameworks are developed and employed for processing image and serial data. They are seldom used in censored and structured medical clinical data. In this study, three DL frameworks, CNN, LSTM, and Resnet, are adapted to process this sort of data and predicting the outcomes of IS. The structure of the adapted CNN is similar to LeNet-5 (LeCun et al., 1998) with an added one-dimensional convolutional layer as the first convolutional layer. The used LSTM and Resnet are also added to a one-dimensional convolutional layer as the first layer. In this way, these DL frameworks can admit and process tabulated data, such as structured medical data. CNN attains the accuracy of 0.83 when trained with less epochs, but it gets less f1-score (Figure 3). This suggested that it doesn’t work well in multiclassification task, so does LSTM (Figure 4). After eliminating the 5 least important features and after trained with more epochs (100 epochs), Resnet gets a higher weighted average f1-score (Figure 15). The first reason is that the left 44 features are more important to the outcomes of IS. The second reason is that Resnet is a fairly complex DL framework. It adopts residual shortcut connection to overcome degradation problems. When trained appropriately Resnet can capture some intrinsic qualities of the tabulated data and work better in a multiclassification task. In this study, the used Resnet is similar to Resnet18 which possesses fewer layers. Next, deeper Resnet framework and more powerful computing workstations will be adopted to study this issue.

To investigate the predicting capability of DL in the IST dataset, the performances of classic ML algorithms are compared to them. The results show that DL doesn’t surpass ML. Resnet raises f1-score after 100 epochs training with the selected 44 features. After eliminating the 5 least important features, the DF and RF raise the f1-score a little and both attain the accuracy of 0.83 (Figures 12, 13). And moderate f1-scores are attained in previous training and test (Figures 6, 7). This means the left 44 features are more important and the 2 ML classifiers are robust to be used in this sort of data. The newly proposed DF is used to be compared with DL frameworks. In our experiments, DF doesn’t achieve highly competitive performance to deep neural networks, although the training time cost of DF is smaller than that of deep learning. The reason of this lies in that the used features were collected in the early 1990s. Some important features may be neglected, and this reduces the predicting ability of ML and DL. Next, deeper DL frameworks will be adopted to investigate the performance of them. Furthermore, some new features and variables will be collected to enhance the performance of the machine learning and deep learning approaches.

## Data Availability

The original contributions presented in the study are included in the article/Supplementary Material, and further inquiries can be directed to the corresponding author.
