# Supplementary material for: Predicting Ischemic Stroke Outcome Using Deep Learning Approaches
Source: Front Genet. 2022 Jan 24;12:827522. doi: 10.3389/fgene.2021.827522 (PMC8818957; doi:10.3389/fgene.2021.827522)
Supplement: Supplementary file 1 [file DataSheet2.PDF]

Supplementary materials

	precision	recall	f1-score	support
0	1.00	1.00	1.00	849
1	0.82	0.85	0.84	2540
2	0.40	0.35	0.37	712
accuracy			0.79	4101
macro avg	0.74	0.73	0.74	4101
weighted avg	0.78	0.79	0.79	4101

Fig. 1. Performance of Resnet with 44 features after 500 epochs of training.

	precision	recall	f1-score	support
0	1.00	1.00	1.00	849
1	0.98	0.10	0.18	2540
2	0.24	0.99	0.38	712
accuracy			0.44	4101
macro avg	0.74	0.70	0.52	4101
weighted avg	0.85	0.44	0.39	4101

Fig. 2. Performance of AdaBoost.

	precision	recall	f1-score	support
0	1.00	0.85	0.92	849
1	0.78	0.65	0.71	2540
2	0.28	0.50	0.36	712
accuracy			0.66	4101
macro avg	0.69	0.67	0.66	4101
weighted avg	0.74	0.66	0.69	4101

Fig. 3. Performance of MultinomialNB.

	precision	recall	f1-score	support
0	1.00	1.00	1.00	849
1	0.98	0.10	0.18	2540
2	0.24	0.99	0.38	712
accuracy			0.44	4101
macro avg	0.74	0.70	0.52	4101
weighted avg	0.85	0.44	0.39	4101

Fig. 4. Performance of AdaBoost with 44 features.

	precision	recall	f1-score	support
0	1.00	0.85	0.92	849
1	0.78	0.65	0.71	2540
2	0.28	0.50	0.36	712
accuracy			0.66	4101
macro avg	0.69	0.67	0.66	4101
weighted avg	0.74	0.66	0.69	4101

Fig. 5. Performance of MultinomialNB with 44 features.

	precision	recall	f1-score	support
0	1.00	1.00	1.00	849
1	0.81	0.93	0.87	2540
2	0.49	0.25	0.33	712
accuracy			0.82	4101
macro avg	0.77	0.72	0.73	4101
weighted avg	0.80	0.82	0.80	4101

Fig. 6. Performance of CNN with 44 features after 50 epochs training.

	precision	recall	f1-score	support
0	0.99	1.00	0.99	849
1	0.81	0.95	0.87	2540
2	0.52	0.19	0.27	712
accuracy			0.83	4101
macro avg	0.77	0.71	0.71	4101
weighted avg	0.79	0.83	0.79	4101

Fig. 7. Performance of CNN with 44 features after 100 epochs training.

	precision	recall	f1-score	support
0	1.00	0.94	0.97	849
1	0.77	1.00	0.87	2540
2	0.67	0.01	0.03	712
accuracy			0.81	4101
macro avg	0.81	0.65	0.62	4101
weighted avg	0.80	0.81	0.74	4101

Fig. 8. Performance of LSTM with 44 features after 50 epochs training.

	precision	recall	f1-score	support
0	1.00	1.00	1.00	849
1	0.80	0.95	0.87	2540
2	0.49	0.17	0.25	712
accuracy			0.82	4101
macro avg	0.76	0.70	0.71	4101
weighted avg	0.79	0.82	0.79	4101

Fig. 9. Performance of LSTM with 44 features after 100 epochs training.

	precision	recall	f1-score	support
0	1.00	1.00	1.00	849
1	0.81	0.93	0.87	2540
2	0.49	0.24	0.32	712
accuracy			0.82	4101
macro avg	0.76	0.72	0.73	4101
weighted avg	0.79	0.82	0.80	4101

Fig. 10. Performance of Resnet with 44 features after 50 epochs training.
