# Supplementary material for: Predicting Ischemic Stroke Outcome Using Deep Learning Approaches
Source: Front Genet. 2022 Jan 24;12:827522. doi: 10.3389/fgene.2021.827522 (PMC8818957; doi:10.3389/fgene.2021.827522)
Supplement: Supplementary file 2 [file DataSheet1.PDF]

Table 2 - Variables names and comments.

**Randomisation data**	
HOSPNUM	Hospital number
RDELAY	Delay between stroke and randomisation in hours
RCONSC	Conscious state at randomisation (F - fully alert, D - drowsy, U - unconscious)
SEX	M=male; F=female
AGE	Age in years
RSLEEP	Symptoms noted on waking (Y/N)
RATRIAL	Atrial fibrillation (Y/N); not coded for pilot phase - 984 patients
RCT	CT before randomisation (Y/N)
RVISINF	Infarct visible on CT (Y/N)
RHEP24	Heparin within 24 hours prior to randomisation (Y/N)
RASP3	Aspirin within 3 days prior to randomisation (Y/N)
RSBP	Systolic blood pressure at randomisation (mmHg)
RDEF1	Face deficit (Y/N/C=can't assess)
RDEF2	Arm/hand deficit (Y/N/C=can't assess)
RDEF3	Leg/foot deficit (Y/N/C=can't assess)
RDEF4	Dysphasia (Y/N/C=can't assess)
RDEF5	Hemianopia (Y/N/C=can't assess)
RDEF6	Visuospatial disorder (Y/N/C=can't assess)
RDEF7	Brainstem/cerebellar signs (Y/N/C=can't assess)
RDEF8	Other deficit (Y/N/C=can't assess)
STYPE	Stroke subtype (TACS/PACS/POCS/LACS/other
RTIME	Time of randomisation (Oxford)
RDATE	Date of randomisation
HOURLOCAL	Local time – hours
MINLOCAL	Local time – minutes
DAYLOCAL	Estimate of local day of week (assuming RDATE is Oxford)
RXASP	Trial aspirin allocated (Y/N)
RXHEP	Trial heparin allocated (M/L/N) [M is coded as H=high in pilot]
**Data collected on 14 day/discharge form about treatments given in hospital**	
DASP14	Aspirin given for 14 days or till death or discharge (Y/N)
DASPLT	Discharged on long term aspirin (Y/N)
DLH14	Low dose heparin given for 14 days or till death/discharge (Y/N)
DMH14	Medium dose heparin given for 14 days or till death/discharge (Y/N)
DHH14	Medium dose heparin given for 14 days etc in pilot (combine with above)
ONDRUG	Estimate of time in days on trial treatment
DSCH	Non trial subcutaneous heparin (Y/N)
DIVH	Non trial intravenous heparin (Y/N)
DAP	Non trial antiplatelet drug (Y/N)
DOAC	Other anticoagulants (Y/N)
DGORM	Glycerol or manitol (Y/N)
DSTER	Steroids (Y/N)
DCAA	Calcium antagonists (Y/N)
DHAEMD	Haemodilution (Y/N)

DCAREND	Carotid surgery (Y/N)
DTHROMB	Thrombolysis (Y/N)
DMAJNCH	Major non-cerebral haemorrhage (Y/N)
DMAJNCHD	Date of above (yyyy/mm/dd)
DMAJNCHX	Comment on above
DSIDE	Other side effect (Y/N)
DSIDED	Date of above (yyyy/mm/dd)
DSIDEX	Comment on above
**Final diagnosis of initial event**	
DDIAGISC	Ischaemic stroke
DDIAGHA	Haemorrhagic stroke
DDIAGUN	Indeterminate stroke
DNOSTRK	Not a stroke
DNOSTRKX	Comment on above
**Recurrent stroke within 14 days**	
DRSISC	Ischaemic recurrent stroke
DRSISCD	Date of above (yyyy/mm/dd)
DRSH	Haemorrhagic stroke
DRSHD	Date of above (yyyy/mm/dd)
DRSUNK	Unknown type
DRSUNKD	Date of above (yyyy/mm/dd)
**Other events within 14 days**	
DPE	Pulmonary embolism
DPED	Date of above (yyyy/mm/dd)
DALIVE	Discharged alive from hospital
DALIVED	Date of above (yyyy/mm/dd)
DPLACE	Discharge destination (A-Home/B-Relatives home/C-Residential care/D-Nursing home/E-Other hospital departments/U-Unknown)
DDEAD	Dead on discharge form
DDEADD	Date of above (yyyy/mm/dd); NOTE: this death is not necessarily within 14 days of randomisation
DDEADC	Cause of death (1-Initial stroke/2-Recurrent stroke (ischaemic or unknown)/3-Recurrent stroke (haemorrhagic)/4-Pneumonia/5-Coronary heart disease/6-Pulmonary embolism/7-Other vascular or unknown/8-Non-vascular/0-unknown)
DDEADX	Comment on death
**Data collected at 6 months**	
FMETHOD	Method of 6 month follow-up (T/P)
FSOURCE	Source of 6 month data
FDEAD	Dead at six month follow-up (Y/N)
FLASTD	Date of last contact
FDEADD	Date of death; NOTE: this death is not necessarily within 6 months of randomisation

FDEADC	Cause of death (1-Initial stroke/2-Recurrent stroke (ischaemic or unknown)/3-Recurrent stroke (haemorrhagic)/4-Pneumonia/5-Coronary heart disease/6-Pulmonary embolism/7-Other vascular or unknown/8-Non-vascular/0-unknown)
FDEADX	Comment on death
FRECOVER	Fully recovered at 6 month follow-up (Y/N)
FDENNIS	Dependent at 6 month follow-up (Y/N)
FPLACE	Place of residance at 6 month follow-up (A-Home/B-Relatives home/C-Residential care/D-Nursing home/E-Other hospital departments/U-Unknown)
FAP	On antiplatelet drugs
FOAC	On anticoagulants
**Other data and derived variables**	
FU1_RECD	Date discharge form received
FU2_DONE	Date 6 month follow-up done
COUNTRY	Abbreviated country code
CNTRYNUM	Country code
FU1_COMP	Date discharge form completed
NCCODE	Coding of compliance (see Table 3)
CMPLASP	Compliant for aspirin
CMPLHEP	Compliant for heparin
ID	Indicator variable for death (1=died; 0=did not die)
TD	Time of death or censoring in days
EXPDD	Predicted probability of death/dependence at 6 month
EXPD6	Predicted probability of death at 6 month
EXPD14	Predicted probability of death at 14 days
SET14D	Know to be dead or alive at 14 days (1=Yes, 0=No); this does not necessarily mean that we know outcome at 6 monts – see OCCODE for this
ID14	Indicator of death at 14 days
OCCODE	Six month outcome (1-dead/2-dependent/3-not recovered/4-recovered/8 or 9 – missing status
SETASPLT	Patient on long term aspirin at 6 months
**Indicator variables for specific causes of death**	
DEAD1	Initial stroke
DEAD2	Reccurent ischaemic/unknown stroke
DEAD3	Reccurent haemorrhagic stroke
DEAD4	Pneumonia
DEAD5	Coronary heart disease
DEAD6	Pulmonary embolism
DEAD7	Other vascular or unknown
DEAD8	Non vascular
H14	Cerebral bleed/heamorrhagic stroke within 14 days; this is slightly wider definition than DRSH an is used for analysis of cerebral bleeds
ISC14	Indicator of ischaemic stroke within 14 days
NK14	Indicator of indeterminate stroke within 14 days
STR14	Indicator of any stroke within 14 days

HTI14	Indicator of haemorrhagic transformation within 14 days
MI14	Indicator of myocardial infarction within 14 days
PE14	Indicator of pulmonary embolism within 14 days
DVT14	Indicator of deep vein thrombosis on discharge form
TRAN14	Indicator of major non-cerebral bleed within 14 days
NCB14	Indicator of any non-cerebral bleed within 14 days
TICH	Time to cerebral bleed (within 14 days)
TMAJH	Time to major non-cerebral bleed (within 14 days)
